# HIV and Syphilis Testing Preferences among Men Who Have Sex with Men in South China: A Qualitative Analysis to Inform Sexual Health Services

**DOI:** 10.1371/journal.pone.0124161

**Published:** 2015-04-13

**Authors:** Cedric H. Bien, Kathryn E. Muessig, Ramon Lee, Elaine J. Lo, Li Gang Yang, Bin Yang, Rosanna W. Peeling, Joseph D. Tucker

**Affiliations:** 1 University of North Carolina-Project China, Guangzhou, China; 2 Mount Sinai School of Medicine, New York, United States of America; 3 Gillings School of Global Public Health, University of North Carolina, Chapel Hill, United States of America; 4 Harvard Medical School, Boston, United States of America; 5 Guangdong Provincial STD Control Center, Guangzhou, China; 6 London School of Hygiene and Tropical Medicine, London, United Kingdom; 7 School of Medicine, University of North Carolina, Chapel Hill, United States of America; Duke University Medical Center, UNITED STATES

## Abstract

**Background:**

Health services for men who have sex with men (MSM) are inadequate in many areas around the world. HIV and syphilis test uptake remain suboptimal among MSM in China and many other regions. To inform the development of more comprehensive sexually transmitted disease (STD) testing programs among MSM, we collected descriptive data on MSM testing practices and preferences.

**Methods:**

MSM in two large urban Chinese cities were recruited through community-based organizations and clinics to participate in semi-structured interviews. We purposively sampled MSM across a range of sociodemographic characteristics and testing history, and assessed preferences for HIV and syphilis testing in the context of facilitators and barriers to testing and previous testing experiences. Each interview transcript was coded and thematically analyzed using Atlas.ti 7.0.

**Results:**

35 MSM were interviewed. Confidentiality and privacy were the most important factors influencing participants’ decisions about whether and where to get tested. Men preferred rapid testing (results available within 30 minutes) compared to conventional tests where results take several hours or days to return. Participants described concerns about quality and accuracy of rapid tests offered in non-clinical settings such as community-based organizations. Men preferred testing service providers who were MSM-friendly, non-discriminatory, and medically trained. Preferred service center environments included: convenient but discrete location, MSM-friendly atmosphere, and clean/standard medical facilities.

**Conclusion:**

Our data highlight the need for HIV/syphilis testing services that are confidential and inclusive of MSM. Rapid testing in decentralized (i.e. peripheral health facilities and community-level, non-clinical venues) settings provides an opportunity to reach individuals who have not been tested before, but must be accompanied by quality assurance systems and technical competence. Implementation research could further evaluate HIV/syphilis testing programs responsive to MSM preferences.

**Short Summary:**

A qualitative study of MSM in South China found that men preferred rapid STD testing at MSM-focused test centers, but were concerned about test quality assurance and confidentiality.

## Introduction

Worldwide, men who have sex with men (MSM) face unique health care challenges exacerbated by persistent stigmatization and discrimination [1]. Following the Institute of Medicine’s 2011 report on lesbian, gay, bisexual and transgender health, renewed focus has been placed on expanding health services research among MSM [2]. Sexual health is a particularly significant issue for MSM because of high prevalence of HIV and other sexually transmitted diseases (STDs) coupled with numerous barriers to optimal care and health services [3,4]. Although some high-income countries now have well-developed MSM sexual health services, MSM remain under-served in many low- and middle-income countries where cultural, political and economic contexts limit HIV/STD surveillance and health programs [3,5].

In China, recent Centers for Disease Control (CDC) efforts have expanded HIV/STD surveillance, but sexual health service uptake among MSM remains low [6]. An estimated 53% of MSM in China have never tested for HIV and 62% of MSM have not received HIV testing in the past year [7]. The prevalence of HIV and other STD co-infection is also high among MSM in China [8,9]. Uncertainty about conventional HIV testing sites, stigma, and fear of discrimination prevent many Chinese MSM from testing [10–14]. In addition, many testing staff in China are inadequately trained to engage and retain MSM in care [15]. A significant proportion of MSM in China do not return for their screening test results or confirmatory tests [16,17], which are often unavailable until one to three days after testing.

Decentralized HIV/STD health care services—defined as testing, counseling or care services provided in peripheral health facilities or community-level facilities and commercial venues (e.g. bars, saunas) outside of hospitals and government-run centers—are facilitated by new point-of-care rapid test technologies for HIV, syphilis, and Hepatitis C [18]. These sexual health services are evolving to better serve the needs of MSM and can be specifically tailored to reach high-risk MSM [19] through organizations that can offer flexibility in test environment, location, and hours of operation. Although studies among MSM in high-income countries have shown that point-of-care tests are preferred over conventional tests [20,21], the introduction of point-of-care testing among MSM in China has been relatively recent [22,23].

Across China, both conventional hospital-based testing and decentralized testing services are becoming more available to MSM [22,24]. Given continued limited uptake of these services, a better understanding of MSM test preferences is needed. This study aims to expand our understanding of current MSM sexual health services in South China, a setting with an estimated HIV prevalence of 5% and syphilis prevalence of 17% among MSM [25]. We focus on HIV and syphilis testing because, in China, point-of-care tests are available and commonly used for these infections. This study investigates MSM preferences for HIV and syphilis testing in order to enhance MSM sexual health services.

## Methods

Adult MSM were recruited from two major cities in South China. In order to capture a wide range of MSM testing experiences and preferences, we purposively sampled men who represented a diversity of HIV and syphilis lifetime testing histories, ages, sociodemographics, sexual self-identity, and marital status. We intentionally sampled men who had never tested for HIV and syphilis, tested only once, and tested multiple times in order to understand factors influencing participants’ decisions about testing. Our recruitment was conducted in two types of community-based locations: 1) a local voluntary counseling and testing (VCT) clinic site in which MSM can book appointments online or in-person; and 2) a local CBO that has long-standing ties to the MSM community and promotes HIV testing. Purposive sampling was accomplished with the assistance of staff at these organizations who have established trusting relationships with their patrons. Staff also used their organizations’ web-based contact networks for recruitment. The staff at the clinic and the CBO referred interested individuals to a research assistant. Eligibility criteria included self-reported ever having sex with another man and being 16 years of age or older (age of legal consent in China).

We conducted semi-structured, one-on-one interviews using an interview guide consisting of questions about HIV/syphilis testing: testing experiences and preferences, stigma, facilitators and barriers to testing, and perceptions and experiences with MSM-focused organizations and services (S1 Data). The number of interviews needed to reach thematic saturation was estimated based on the focused nature of our research questions around HIV and syphilis testing preferences, and the need to sufficiently describe variation in patterns of MSM testing behavior [26].

Interviews were conducted by trained Chinese-American bilingual speakers (one Mandarin-English, one Cantonese-English) at a convenient, private location and time of the participants’ choosing. As many Chinese in this area are bi- or tri-lingual, participants were given a choice for their preferred interview language (Mandarin, Cantonese, or English). Interviews were recorded with permission and participants were offered either a phone card or shopping card worth approximately nine USD as remuneration. Interviews were transcribed and translated by native Cantonese and Mandarin speakers, and then checked for accuracy and quality by separate bilingual study personnel.

We used a Framework Analysis to accommodate *a priori* and emergent themes [27]. Based on these themes, the research team developed a codebook that defined a list of thematic codes and example interview quotes for each code. Two coders used this codebook with Atlas.ti 7.0 qualitative data analysis software to attach codes to relevant blocks of text, and a third analyst then reviewed inter-coder consistency.

There are no direct translations of the concept terms “gay-friendly,” “MSM-friendly,” or “MSM-tailored” in Chinese. During interviews, men most commonly described these concepts as “for people like us” or by providing examples from their experiences. For simplicity, we use the term “MSM-friendly” to loosely describe services, providers, and testing environments that have been designed to be inclusive of MSM, or exclusively for MSM.

This study was conducted according to the principles expressed in the Declaration of Helsinki, and was approved by the Institutional Review Boards of the Guangdong Provincial STD Control Center, the London School of Hygiene and Tropical Medicine, and the University of North Carolina at Chapel Hill. These IRBs determined the risks of study participation as minimal and approved a verbal informed consent protocol. Each interviewer and a witness signed a written statement documenting the time and date of each interview. No next of kin, caretakers, or guardians were interviewed on behalf of minors. We followed the consolidated criteria for reporting qualitative research (COREQ) (S1 Table).

## Results

We interviewed 35 MSM (Table 1). Participants ranged in age from 18 to 48 years old, with the majority of men between 26 and 40 years old. The majority reported current employment (24/35), and most had completed high school (33/35). Regarding sexual orientation, 28 self-identified as gay, four as bisexual, and three were not sure or did not wish to report this information. A majority of interviewees had tested multiple times for HIV or syphilis (21/35), while seven men had completed their first test on the day of their interview, and seven had never been tested.

**Table 1:** Demographic characteristics of MSM study participants in two Southern Chinese cities.

**Characteristic**	**No. of MSM N = 35**	**(%)**
**Age (years)**		
16–25	13	(37)
26–40	20	(57)
Over 40	2	(6)
**Testing status**		
Refused testing/never tested	7	(20)
First time testing	7	(20)
More than one test in the past	21	(60)
**Education**		
Less than high school	2	(6)
Completed high school	8	(23)
More than high school	25	(71)
**Hometown location**		
Guangdong Province	12	(34)
Hong Kong	8	(23)
Other	11	(31)
Not reported	4	(11)
**Employment status**		
Currently working	24	(68)
Student	7	(20)
Not currently working	3	(9)
Not reported	1	(3)
**Race/ethnicity**		
Han	27	(77)
Other	3	(9)
Not reported	5	(14)
**Sexual orientation**		
Gay	28	(80)
Bisexual	4	(11)
Not sure	1	(3)
Not reported	2	(6)
**Marital status**		
Unmarried	28	(80)
Widowed/divorced/separated	1	(3)
Engaged/Married	5	(14)
Not reported	1	(3)

Men described what they felt were the most important elements that influenced their decisions about whether and where to get HIV/syphilis testing. While there was overlap across these elements, we present them as four interrelated themes below: (1) a preference for rapid testing services; (2) a desire for increased confidentiality of testing services; (3) a desire for increased sensitivity of service providers; and (4) the importance of a relaxed yet “professional” testing environment.

### Preference for rapid testing services

Most MSM preferred rapid HIV and syphilis tests compared with conventional laboratory-based tests. Aside from the convenience of receiving results within 30 minutes as compared to hours or days, MSM also cited other advantages to rapid tests including decreased anxiety over results and increased confidentiality. One man stated:

My first thought [after testing] was… nervousness? But now they have those rapid test kits, so I think it’s okay, because you don’t have to wait for a week or so, so the nervous period can be shortened. (#30, age 39, multiple-time tester)

Rapid testing also enables the possibility of self-testing, allowing men to take tests alone on their own terms with or without supervision. The option of self-testing may facilitate testing among men who are reluctant to attend testing centers:

I guess I would self-test. I can do the testing myself, it’s much more private… Maybe I won’t go to test [at a testing facility] unless I have some symptoms or illness. (#21, age 26, never tested)

Some men described a trade-off between knowing results earlier and a perceived decrease in test accuracy:

Well I believe there are some errors for rapid tests, which everyone knows. And when I do the test, I will tell the staff that I know it has errors. But for regular testing, I will definitely do [the rapid testing]. (#07, age 33, multiple-time tester)

### Desire for increased confidentiality of testing services

Men reported that some clinic services lacked sufficient protocols to protect patient confidentiality. This response was typical of men’s complaints about testing facility practices:

We have had the experience of seeing doctors with [other] patients, and this is a real case, where the hospital uses a broadcasting system to call patients’ names when one’s turn is coming. (#07, age 33, multiple-time tester)

In addition, many participants did not trust providers in hospital-based and CDC testing settings to protect their confidentiality. As explained by one man:

I think that their medical staff [at CDC testing centers], even though they are professional, they don't have good attitudes. Apart from that, I tend to wonder if they would disclose [information] recklessly. Therefore I worry. (#29, age 21, multiple-time tester)

There were several aspects of testing facilities that men felt could promote confidentiality of testing services including discrete clinic locations and anonymous testing services. Participants universally preferred to leave pseudonyms or phone numbers when registering for testing:

I think in the hospital my privacy was not as well protected as in here [MSM community-based organization]. [At the hospital] I must show my identity card or other certificates. But here, I can just write down my phone number. Nothing more is required. (#03, age 27, multiple-time tester)

### Desire for increased sensitivity of service providers

A few participants stated that many hospital or CDC clinics lacked adequate staff training for providing MSM-specific care. Men preferred test service providers that offered counseling and testing that was sensitive to their needs as gay men. Many participants commented that they felt discriminated against by service providers within the formal medical system such as hospital and CDC testing centers. A number of men described experiencing stigma against MSM and stigma related to HIV testing among service providers, which discouraged them from disclosing their sexual identity to clinic staff in formal medical settings or from future testing:

I felt discrimination from all kinds of people at other places…like the CDC…The way they spoke to me and the way they looked at me. (#10, age 27, multiple-time tester)

I mean if you go to the normal medical center, you cannot talk about your real reason to do the test. You may hide the true reason, that you had sex with a man. [At an MSM-tailored service provider] You just tell your true story… Yes, I mean they can provide better service and treat this kind of people [MSM] better. (#13, age 33, multiple-time tester)

Many participants also highlighted the importance of supportive counselling services provided at MSM-focused testing centers. As one participant stated:

When you have blood drawn [at this MSM-focused organization], there is a volunteer who comes and talks to you. You can consult a lot of things with him or her, which makes you feel good and comfortable, so that you may get tested here again in the future. (#07, age 33, multiple-time tester)

### The importance of a relaxed yet “professional” testing environment

Participants also described specific preferences for test environments and facilities. Most men preferred testing environments that were relaxed and gay-friendly:

The first factor [in choosing where to test] is atmosphere. An atmosphere like what we are having now, relaxed and informal. (#02, age 24, multiple-time tester)

I think gay-friendly is the most important thing—and I can talk to people, you know, talk to you. You can’t find this service in the hospital. (#05, age 29, first time tester)

Although a number of men stressed the importance of an “informal” or relaxed environment, a clean, professional atmosphere was also important:

Personally I like to be clean. I think if a center… If a testing center is very messy, you will feel very uncomfortable and you will never want to come back. (#12, age 28, multiple-time tester)

Along with a professional atmosphere, several men spoke of the need for test facilities to have official recognition:

They [CBOs] are not official, or recognized by law… so, I can’t *really truly* trust them, although maybe they can help me anyway… I just trust the official places, official organizations. I think they provide me with all the standard and good services. I trust them. (#02, age 24, multiple-time tester)

Men were aware that HIV testing is now being increasingly offered as point-of-care, venue-based testing where MSM socialize and find sexual partners. In our sample, some men expressed concern regarding the professionalism of MSM venue-based testing:

I would maybe go to entertainment venues [for testing]… If they have testing, they must be supported by some professional organization, and not just the gay bar itself, so I’m most concerned about it being professional. (#22, age 28, never tested)

## Discussion

Multi-level factors related to available testing technologies, stigma, service providers, and testing environments contributed to HIV/syphilis testing behaviors and preferences among MSM in this sample. In recent years, various HIV/syphilis testing initiatives for MSM have been piloted in major Chinese cities [10,24,28]. Many of these initiatives have incorporated testing outside of hospital-based facilities, a critical step toward expanding access to sexual health services [29]. This decentralized testing (i.e. provided outside of traditional hospital- and CDC-based settings) is increasingly common in China [30]. In fact, in many areas of China, CBO-based testing may account for over half of all newly identified HIV infections among Chinese MSM [31]. Our research extends previous qualitative literature [32] on MSM sexual health services by focusing on men’s preferences and contextual factors that influence test uptake. Our inclusion of MSM who had never received HIV testing may help better understand barriers to first-time testing.

Rapid tests allow expansion of testing by eliminating the need for an onsite laboratory and making same-day test results available. Studies in high-income nations suggest that expanding rapid testing services among MSM may increase test uptake and frequency [20,21]. Men in our sample preferred rapid testing compared to conventional tests and described advantages such as decreased anxiety, increased convenience and greater confidentiality. However, some participants were skeptical about rapid test accuracy. Additionally, some men were concerned about the quality, cleanliness, and professionalism of both rapid and traditional testing services offered in non-traditional settings such as CBOs, bathhouses, saunas and gay bars. This result is consistent with findings from the U.S. [33] and United Kingdom [34]. Research from low-income countries has shown that decentralized HIV testing may increase false positive testing [35]. Rapid test quality assessment algorithms and training programs have been developed and their implementation is imperative in ensuring test quality in any setting [36,37]. Of note, MSM in this sample were also interested in home-based self-testing. Like venue-based rapid testing, at-home self-testing may also successfully identify a large proportion of undiagnosed Chinese MSM [22]. This practice is growing in popularity in China [22,38] but faces similar concerns from men about accuracy [39]. As China moves toward a model where a significant proportion of all HIV tests are rapid [40] and/or offered at the community-level [31], tailored health communication messaging and pre- and post-test counseling may also need to more proactively address quality assurance and accuracy concerns.

Ensuring confidentiality is also critical for expanding HIV/STD testing among MSM in China [41,42] and globally [20]. In our study, privacy concerns surrounded both the stigma of HIV/STD testing itself, and the implication of HIV/STD testing for disclosure of men’s sexual orientation (to health care professionals as well as other patients). Many MSM in China protect their sexual identity from their families, friends, and coworkers. Thus, HIV testing itself in selected settings could be perceived as a form of disclosure [12]. Our qualitative data reflected these concerns as several MSM described previous testing experiences in which their personal information—such as name, reason for seeking medical care, and test results—was publicly revealed. At-home self-testing offers one potential testing strategy that could address concerns regarding confidentiality and privacy [12], and as discussed above, may require additional messaging and promotion.

Stigma, discrimination, and fear were overarching deterrents many men in this study faced in accessing sexual health services. Similar to results from other countries [34,43,44], fear of stigma and discrimination from providers were a significant barrier to HIV testing for Chinese MSM in this sample. Our data highlight the importance of developing MSM-friendly testing and sexual health services in China. These services will require additional clinical training (e.g. anal and laryngeal swabs; tailored diagnostic interviewing for MSM); sensitivity training (e.g. anti-stigma, anti-discrimination for HIV and sexual orientation); MSM-tailored counseling and testing messages, MSM-relevant risk reduction strategies, and a clean, professional testing environment. A cross-sectional study of STD clinics in South China found that only 32% of clinics reported that any staff had received MSM-related training; even fewer (14%) had sexual health information tailored for MSM [29]. This problem is common in many low- and middle-income nations where HIV/STD services for MSM are under-supported. However, these are also the same settings where increased MSM-friendly clinical training and more active engagement with MSM communities could facilitate test uptake and assuage concerns about confidentiality and stigma.

Several considerations should be kept in mind alongside our findings. First, homosexuality remains a sensitive issue in China, and MSM in our study may have selectively chosen how they discussed certain topics including their motivations for testing as related to HIV/STD high-risk behaviors. We partnered with several CBOs and pilot tested our questions in order to build rapport within these sensitive settings. Men’s willingness to share both positive and negative evaluations of their testing experiences and descriptions of infrequent/never testing suggest a lower chance that social desirability biases affected participants’ responses around these particular issues. Second, our study was not designed to infer relationships between men’s stated testing preferences and their actual testing behaviors. Rather, our goal was to understand how men explained their testing experiences, preferences, and decisions. Further implementation research is needed to gauge how stated preferences are related to actual testing practices. Third, our data makes some broad comparisons between HIV testing sites based on men’s experiences, but the study was not designed as a standardized comparison of all the various testing options available to MSM in China.

The urgency of enhancing HIV and syphilis testing among MSM in China has been recognized by the central government. In 2007, China announced a five-year *Plan for HIV/AIDS Prevention and Control among Men Who Have Sex with Men in China* which included expanded HIV testing as a core component [45]. Three years later, China launched a comprehensive national syphilis control plan that also focused on increasing organizational capacity for testing [46]. Nevertheless, many existing testing services offered in South China are still not well-suited to the unique needs of MSM populations. As advances in diagnostic technologies continue to facilitate expansion of testing options, additional research is needed to engage MSM and maintain high test quality. Interventions relying on social forces [10,24,47] may increase HIV test uptake and decrease test-related stigma and fear. Structural interventions may also be necessary to maintain an environment conducive to HIV/syphilis testing.

## Supporting Information

S1 Data Semi-structured interview guide. (DOC)

S1 Table COREQ checklist for research study. (DOCX)

## References

[1] Beyrer C, Sullivan PS, Sanchez J, Dowdy D, Altman D, et al (2012) A call to action for comprehensive HIV services for men who have sex with men. Lancet 380: 424–438. 10.1016/S0140-6736(12)61022-8 22819663 PMC3805059

[2] Institute of Medicine (2011) The Health of Lesbian, Gay, Bisexual, and Transgender People: Building a Foundation for Understanding. Washington, D.C. National Academies Press. 22013611

[3] Beyrer C, Baral SD, van Griensven F, Goodreau SM, Chariyalertsak S, et al (2012) Global epidemiology of HIV infection in men who have sex with men. Lancet 380: 367–377. 10.1016/S0140-6736(12)60821-6 22819660 PMC3805037

[4] Cochran SD, Mays VM (2011) Sexual orientation and mortality among US men aged 17 to 59 years: results from the National Health and Nutrition Examination Survey III. Am J Public Health 101: 1133–1138. 10.2105/AJPH.2010.300013 21493941 PMC3093261

[5] Baral S, Sifakis F, Cleghorn F, Beyrer C (2007) Elevated Risk for HIV Infection among Men Who Have Sex with Men in Low- and Middle-Income Countries 2000–2006: A Systematic Review. PLoS Med 4: e339 18052602 10.1371/journal.pmed.0040339 PMC2100144

[6] Zeng G, Xiao Y, Xu P, Feng N, Jin CR, Lu F (2009) [Evaluation of effect of community-based HIV/AIDS interventions among men who have sex with men in eighteen cities, China]. Zhonghua Yu Fang Yi Xue Za Zhi, 43: 977–980. 20137519

[7] Zou H, Hu N, Xin Q, Beck J (2012) HIV Testing Among Men Who Have Sex with Men in China: A Systematic Review and Meta-Analysis. AIDS Behav 16: 1717–1728. 22677975 10.1007/s10461-012-0225-y

[8] Yin YP, Chen SC, Wang HC, Wei WH, Wang QQ, et al (2012) Prevalence and risk factors of HSV-2 infection and HSV-2/HIV coinfection in men who have sex with men in China: a multisite cross-sectional study. Sex Transm Dis 39: 354–358. 10.1097/OLQ.0b013e318244aef0 22504598

[9] Chow EP, Wilson DP, Zhang L (2011) HIV and syphilis co-infection increasing among men who have sex with men in China: a systematic review and meta-analysis. PLoS One 6: e22768 10.1371/journal.pone.0022768 21857952 PMC3156129

[10] Zou H, Wu Z, Yu J, Li M, Ablimit M, et al (2013) Internet-facilitated, voluntary counseling and testing (VCT) clinic-based HIV testing among men who have sex with men in China. PLoS One 8: e51919 10.1371/journal.pone.0051919 23418417 PMC3572127

[11] Choi KH, Lui H, Guo Y, Han L, Mandel JS (2006) Lack of HIV testing and awareness of HIV infection among men who have sex with men, Beijing, China. AIDS Educ Prev 18: 33–43. 16539574 10.1521/aeap.2006.18.1.33

[12] Wei C, Yan H, Yang C, Raymond HF, Li J, et al (2013) Accessing HIV testing and treatment among men who have sex with men in China: A qualitative study. AIDS Care 26: 372–378. 10.1080/09540121.2013.824538 23909807 PMC4053186

[13] Li X, Lu H, Ma X, Sun Y, He X, et al (2012) HIV/AIDS-related stigmatizing and discriminatory attitudes and recent HIV testing among men who have sex with men in Beijing. AIDS Behav 16: 499–507. 10.1007/s10461-012-0161-x 22350831 PMC4048063

[14] Zhang L, Xiao Y, Lu R, Wu G, Ding X, et al (2013) Predictors of HIV testing among men who have sex with men in a Large Chinese City. Sex Transm Dis 40: 235–240. 10.1097/OLQ.0b013e31827ca6b9 23403605 PMC3725775

[15] Chai J, Wang D, Zhou M, Xu W, Liang G, et al (2011) Developing and piloting an expert system for better routine voluntary HIV counseling and testing in China: preliminary results and lessons. AIDS Care 24: 424–433. 10.1080/09540121.2011.613907 22149044

[16] Lambdin B, Cai T, Udoh I, Lu L, LU X, et al. (2012) Identifying bottlenecks: loss to follow-up of MSM from HIV testing to treatment in Wuhan, China. XIX International AIDS Conference. Washington, DC, USA: Abstract #LBPE51.

[17] Zhang D, Li C, Meng S, Qi J, Fu X, et al (2014) Attrition of MSM with HIV/AIDS along the continuum of care from screening to CD4 testing in China. AIDS Care 26: 1118–1121. 10.1080/09540121.2014.902420 24684294

[18] Tucker JD, Bien CH, Peeling RW (2013) Point-of-care testing for sexually transmitted infections: recent advances and implications for disease control. Curr Opin Infect Dis 26: 73–79. 10.1097/QCO.0b013e32835c21b0 23242343 PMC3635142

[19] Lorente N, Preau M, Vernay-Vaisse C, Mora M, Blanche J, et al (2013) Expanding Access to Non-Medicalized Community-Based Rapid Testing to Men Who Have Sex with Men: An Urgent HIV Prevention Intervention (The ANRS-DRAG Study). PLoS ONE 8: e61225 10.1371/journal.pone.0061225 23613817 PMC3628708

[20] Cohall A, Dini S, Nye A, Dye B, Neu N, et al (2010) HIV testing preferences among young men of color who have sex with men. Am J Public Health 100: 1961–1966. 10.2105/AJPH.2008.140632 20075330 PMC2936982

[21] Lee D, Fairley C, Cummings R, Bush M, Read T, et al (2010) Men who have sex with men prefer rapid testing for syphilis and may test more frequently using it. Sex Transm Dis 37: 557–558. 20803780 10.1097/olq.0b013e3181d707de

[22] Tao J, Li MY, Qian HZ, Wang LJ, Zhang Z, et al (2014) Home-based HIV testing for men who have sex with men in China: a novel community-based partnership to complement government programs. PLoS One 9: e102812 10.1371/journal.pone.0102812 25051160 PMC4106852

[23] Marley G, Kang D, Wilson EC, Huang T, Qian Y, et al (2014) Introducing rapid oral-fluid HIV testing among high risk populations in Shandong, China: feasibility and challenges. BMC Public Health 14: 422 10.1186/1471-2458-14-422 24884431 PMC4045859

[24] Yan H, Zhang R, Wei C, Li J, Xu J, et al (2014) A peer-led, community-based rapid HIV testing intervention among untested men who have sex with men in China: an operational model for expansion of HIV testing and linkage to care. Sex Transm Infect 90: 388–393. 10.1136/sextrans-2013-051397 24926040

[25] Zhong F, Lin P, Xu H, Wang Y, Wang M, et al (2011) Possible increase in HIV and syphilis prevalence among men who have sex with men in Guangzhou, China: results from a respondent-driven sampling survey. AIDS Behav 15: 1058–1066. 10.1007/s10461-009-9619-x 19826942

[26] Hsieh HF, Shannon SE (2005) Three approaches to qualitative content analysis. Qual Health Res 15: 1277–1288. 16204405 10.1177/1049732305276687

[27] Dixon-Woods M (2011) Using framework-based synthesis for conducting reviews of qualitative studies. BMC Med 9: 39 10.1186/1741-7015-9-39 21492447 PMC3095548

[28] Huang Z, Wang M, Fu L, Fang Y, Hao J, et al (2013) Intervention to increase condom use and HIV testing among men who have sex with men in China: a meta-analysis. AIDS Res Hum Retroviruses 29: 441–448. 10.1089/AID.2012.0151 23083341

[29] Yang L. Barriers to STI clinical services for MSM in South China. October 16, 2012; International Union Against Sexually Transmitted Infections (IUSTI) World Congress, Melbourne, Australia. Abstract #1199, Program pg 135. Available online at: http://www.iusti.org/events/images/IUSTI_2012_Handbook_ONLINE.pdf Accessed 2015 February 14.

[30] Fan EL (2014) HIV testing as prevention among MSM in China: the business of scaling-up. Glob Public Health 9: 85–97. 10.1080/17441692.2014.881520 24498955

[31] Zhang DP, Han L, Li CM, Meng SN, Leng ZW, et al (2013) [The impact of community-based organizations in HIV testing mobilization among men who have sex with men]. Zhonghua Yu Fang Yi Xue Za Zhi 47: 431–434. 23958126

[32] Tucker JD, Muessig KE, Cui R, Bien CH, Lo EJ, et al (2014) Organizational characteristics of HIV/syphilis testing services for men who have sex with men in South China: a social entrepreneurship analysis and implications for creating sustainable service models. BMC Infect Dis 14: 601 10.1186/s12879-014-0601-5 25422065 PMC4247875

[33] Prost A, Chopin M, McOwan A, Elam G, Dodds J, et al (2007) "There is such a thing as asking for trouble": taking rapid HIV testing to gay venues is fraught with challenges. Sex Transm Infect 83: 185–188. 17229791 10.1136/sti.2006.023341 PMC2659088

[34] Prost A, Sseruma WS, Fakoya I, Arthur G, Taegtmeyer M, et al (2007) HIV voluntary counselling and testing for African communities in London: learning from experiences in Kenya. Sex Transm Infect 83: 547–551. 17911136 10.1136/sti.2007.027110 PMC2598659

[35] Shanks L, Klarkowski D, O'Brien DP (2013) False positive HIV diagnoses in resource limited settings: operational lessons learned for HIV programmes. PLoS One 8: e59906 10.1371/journal.pone.0059906 23527284 PMC3603939

[36] Learmonth KM, McPhee DA, Jardine DK, Walker SK, Aye TT, et al (2008) Assessing proficiency of interpretation of rapid human immunodeficiency virus assays in nonlaboratory settings: ensuring quality of testing. J Clin Microbiol 46: 1692–1697. 10.1128/JCM.01761-07 18353938 PMC2395071

[37] Chiu YH, Ong J, Walker S, Kumalawati J, Gartinah T, et al (2011) Photographed rapid HIV test results pilot novel quality assessment and training schemes. PLoS ONE 6: e18294 10.1371/journal.pone.0018294 21483842 PMC3069085

[38] Han L, Bien CH, Wei C, Muessig KE, Yang M, et al (2014) HIV self-testing among online MSM in China: implications for expanding HIV testing among key populations. J Acquir Immune Defic Syndr 67: 216–221. 10.1097/QAI.0000000000000278 24991972 PMC4162828

[39] Xu Y, Zhang Z, Li D, Liu Y, Pan SW, et al (2013) Willingness to use the oral fluid HIV rapid test among men who have sex with men in Beijing, China. PLoS One 8: e64652 10.1371/journal.pone.0064652 23717645 PMC3662656

[40] Zhang D, Qi J, Fu X, Meng S, Li C, et al. (2014) Case finding advantage of rapid tests in community settings: men who have sex with men in 12 program areas in China, 2011. Int J STD AIDS, epub ahead of print, 2014 Jul 15. 10.1177/0956462414542986 25028452

[41] Huang ZJ, He N, Nehl EJ, Zheng T, Smith BD, et al (2012) Social network and other correlates of HIV testing: findings from male sex workers and other MSM in Shanghai, China. AIDS Behav 16: 858–871. 10.1007/s10461-011-0119-4 22223298 PMC8080277

[42] Wei S, Zhang H, Wang J, Song D, Duan Y, et al (2013) HIV and Syphilis Prevalence and Associated Factors Among Young Men Who Have Sex with Men in 4 Cities in China. AIDS Behav 17:1151–1158. 10.1007/s10461-011-0110-0 22198314

[43] Mimiaga MJ, Goldhammer H, Belanoff C, Tetu AM, Mayer KH (2007) Men who have sex with men: perceptions about sexual risk, HIV and sexually transmitted disease testing, and provider communication. Sex Transm Dis 34: 113–119. 16810121 10.1097/01.olq.0000225327.13214.bf

[44] Andrinopoulos K, Hembling J, Guardado ME, de Maria Hernandez F, Nieto AI, et al (2015) Evidence of the Negative Effect of Sexual Minority Stigma on HIV Testing Among MSM and Transgender Women in San Salvador, El Salvador. AIDS Behav 19: 60–71. 10.1007/s10461-014-0813-0 24907779

[45] Wu Z, Wang Y, Mao Y, Sullivan SG, Juniper N, et al (2011) The integration of multiple HIV/AIDS projects into a coordinated national programme in China. Bull World Health Organ 89: 227–233. 10.2471/BLT.10.082552 21379419 PMC3044250

[46] Tucker JD, Yin YP, Wang B, Chen XS, Cohen MS (2011) An expanding syphilis epidemic in China: epidemiology, behavioural risk and control strategies with a focus on low-tier female sex workers and men who have sex with men. Sex Transm Infect 87 Suppl 2: ii16–18. 10.1136/sti.2010.048314 22110145 PMC3306605

[47] Wei C, Muessig KE, Bien C, Yang L, Meng R, et al (2014) Strategies for promoting HIV testing uptake: willingness to receive couple-based and collective HIV testing among a cross-sectional online sample of men who have sex with men in China. Sex Transm Infect 90: 469–474. 10.1136/sextrans-2013-051460 24760266 PMC4151526

