# Supplementary material for: HIV and Syphilis Testing Preferences among Men Who Have Sex with Men in South China: A Qualitative Analysis to Inform Sexual Health Services
Source: PLoS One. 2015 Apr 13;10(4):e0124161. doi: 10.1371/journal.pone.0124161 (PMC4395264; doi:10.1371/journal.pone.0124161)
Supplement: S1 Data — (DOC) [file pone.0124161.s001.doc]

**Supplemental Data 1. Semi-structured interview guide.**

**CBO capacity**

How did you learn about this CBO?

Could you tell me about your experiences with HIV/syphilis testing? Experiences with this CBO?

Do you access services from more than one MSM CBO? [Probe: If yes, please describe the other organizations.]

Could you talk about the reputation about the MSM CBOs in the city? Which is the most trusted MSM CBO in your city?

**Sexual orientation**

Some people think of sexuality as a range or spectrum and everyone describes their own sexuality differently.

How would you describe your sexuality? [e.g. gay, tong.zhi, MSM, bisexual, etc.]

Who have you told about your sexuality?

What was their response?

**HIV and syphilis needs**

What is your first thought when you think about HIV/syphilis testing?

Where would you prefer to get HIV/syphilis testing? [Probe: Could you tell me a little bit about why?]

Where would you prefer to get other sexual health services (e.g. treatment, physical exams)?

Do you think that some clinics or hospitals are more gay-friendly than others?

**Multi-time testers only:**

Can you describe some of your testing experiences?

When you were deciding where to go for testing, how did you make the decision about where to go?

[If they have had experiences testing in different settings – hospital vs. other setting: How do you compare these experiences?]

Do you remember how you were feeling at those times?

How did that compare to your testing experience today?

Do you have plans to get tested in the future?

What are your concerns about going to get tested in the future?

**First-time testers only:**

Today was your first time having an HIV/syphilis test.

How did you make the decision about coming here for testing?

Can you describe how you were feeling before you came in for the test?

How was your testing experience today?

How did your experience compare to your expectations?

What are your concerns about going to get tested again in the future?

**Never testers only**:

You mentioned earlier that you have never had an HIV/syphilis test.

What are some of your concerns about having an HIV/syphilis test?

**Facilitators and barriers to testing**

What would make it easier for you to get HIV/syphilis testing?

What would make it easier for you to visit clinical versus non-clinical sites for HIV/syphilis testing?

What would make it more difficult for you to get HIV/syphilis testing?

What would make it difficult for you to visit clinical versus non-clinical sites for HIV/syphilis testing?

To you, what is the most important thing about an HIV/testing site? (e.g. cost, environment, confidentiality, convenience, MSM-friendly)

**Sexual risk behaviors and condom use**

In your current situation, what do you think your risk is for getting HIV or syphilis? Why?

When you are together with your partner/boyfriend, what is your current situation for using condoms?

[Use or not use response] How do you make the decision about whether or not to use condoms?

[If they use them] Where do you generally get condoms? [Probe: free or buy?]

[If they use them] What do you think about getting free condoms versus buying them yourself?

**Opinions of others [Ask interviewee about their peer, manager, family member, friend]**

Who would most influence your decision to get tested for HIV or STIs?

Who would most influence your decision to attend a clinic?

“**Face” and STI testing**

Do you think you would lose face by going to a clinic for HIV/syphilis testing?

Do you think you would lose face by having a positive HIV or syphilis test?

If you suspected you had HIV or syphilis, who would you tell? How did you choose this person?

If you got HIV or syphilis, what kind of response would your family or friends have?

**Health Communication**

How do you feel about the HIV/STI doctors you have met in the past?

How trustworthy do you think HIV/STI doctors are? What would make you trust one doctor more than others?

How do you feel about the confidentiality of HIV/STI testing results?

What do you think about the confidentiality of your results?

What do you think about the validity of HIV or syphilis tests?

We are also interested in new ways of communicating health information and testing.

What kind of mobile phone do you currently use? (Phone type, capabilities, phone plan, type of phone platform, whether phone has GPS capability, whether plan has unlimited texting, whether phone has apps).

Have you ever used your phone to look up information about sexual health? Can you give an example?

What are your thoughts (positive, negative, experiences) about using mobile technologies as part of your health care? [Probe for concerns about confidentiality with: receiving calls/texts/reminders from health care provider; QQ chat with a sexual health care expert; Website with information about staying healthy, sexual health, HIV; Games for learning about sexual health; Mobile phone apps for sexual health]

**Financing**

Have you ever had to pay for HIV/STI testing? If yes, where did you have to pay? What did you think of this cost?

Has there ever been a time when you wanted/needed sexual health services but could not pay for them? Please describe.

How do you think other MSM you know would feel about paying for testing?

**Marketing**

Do you buy products or services that are targeted to MSM?

Do you read MSM-focused magazines, websites, or social media groups?

If an advertisement selling a product depicts MSM life, does that make you more or less willing to buy it?

**Demographics**

What is your age, ethnicity, education background, employment, marital status?
